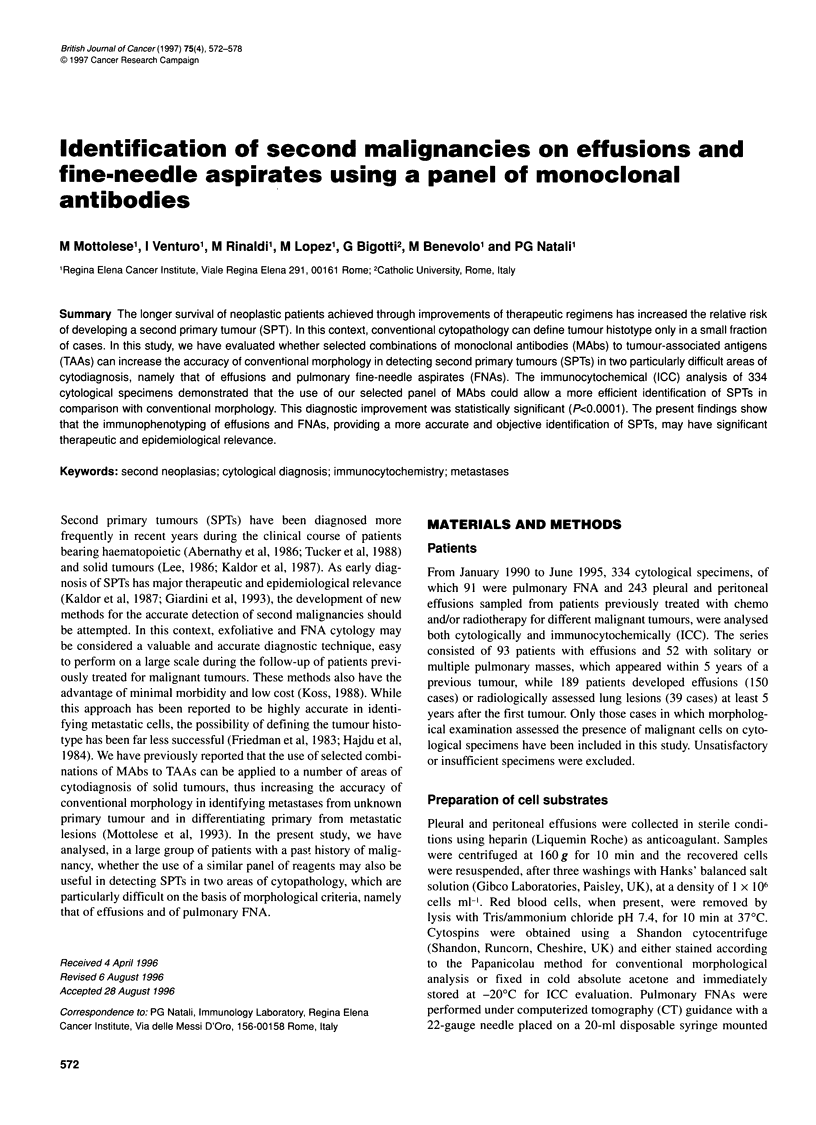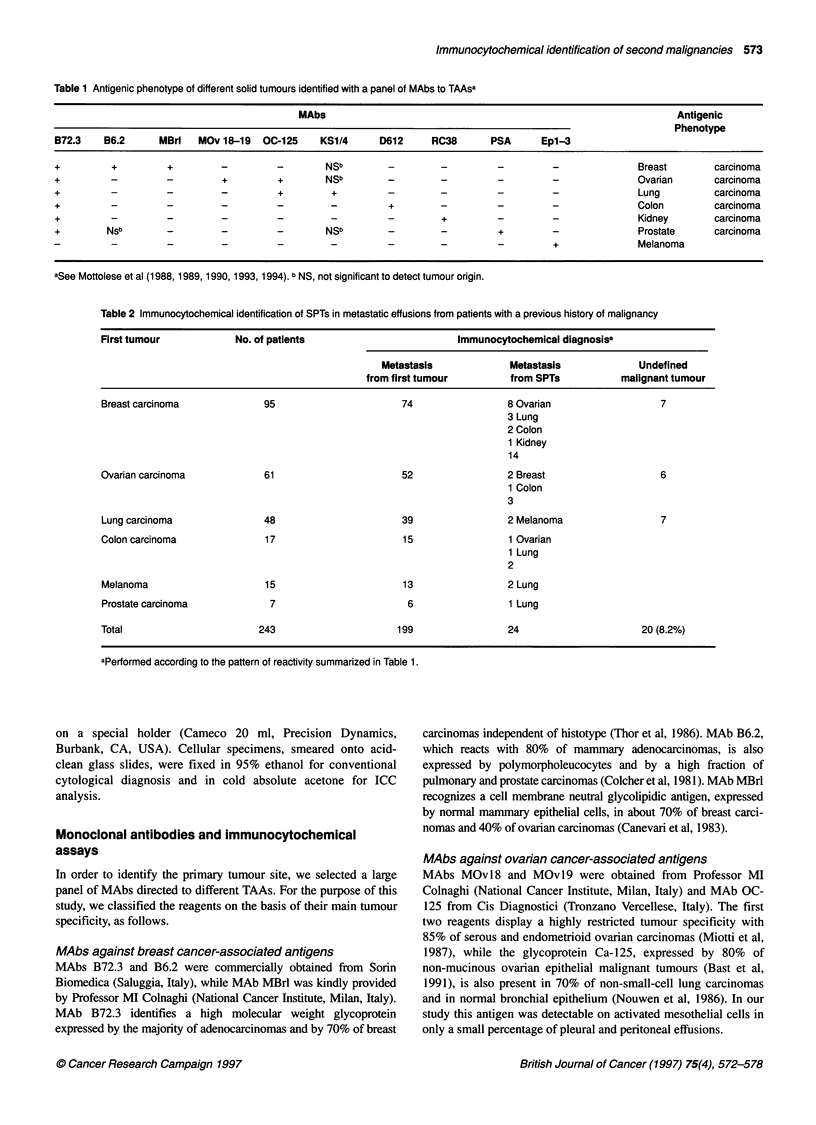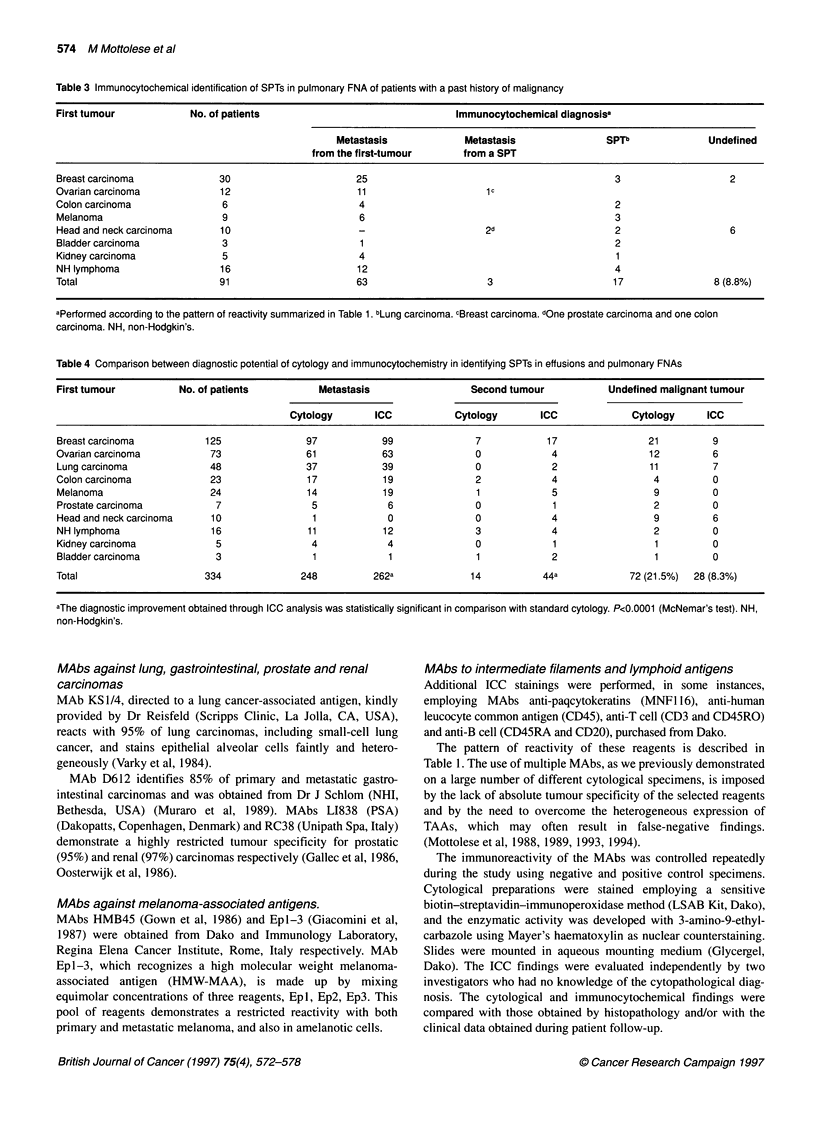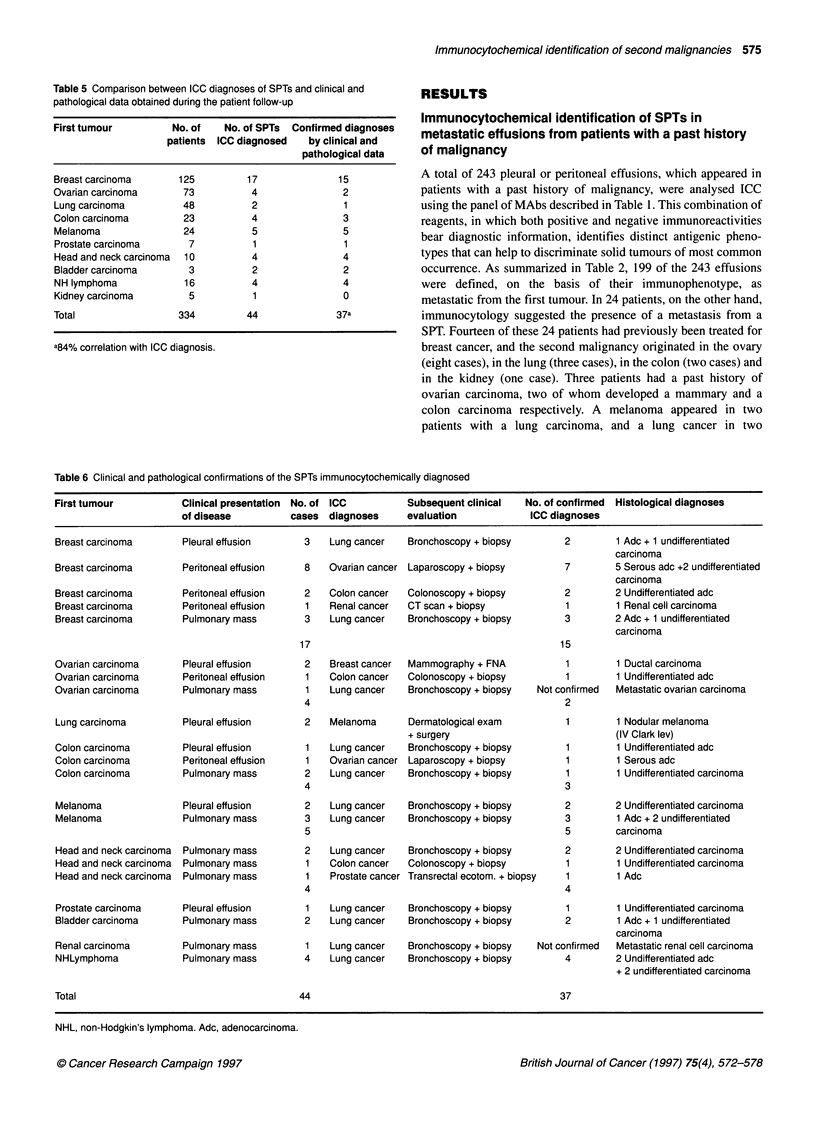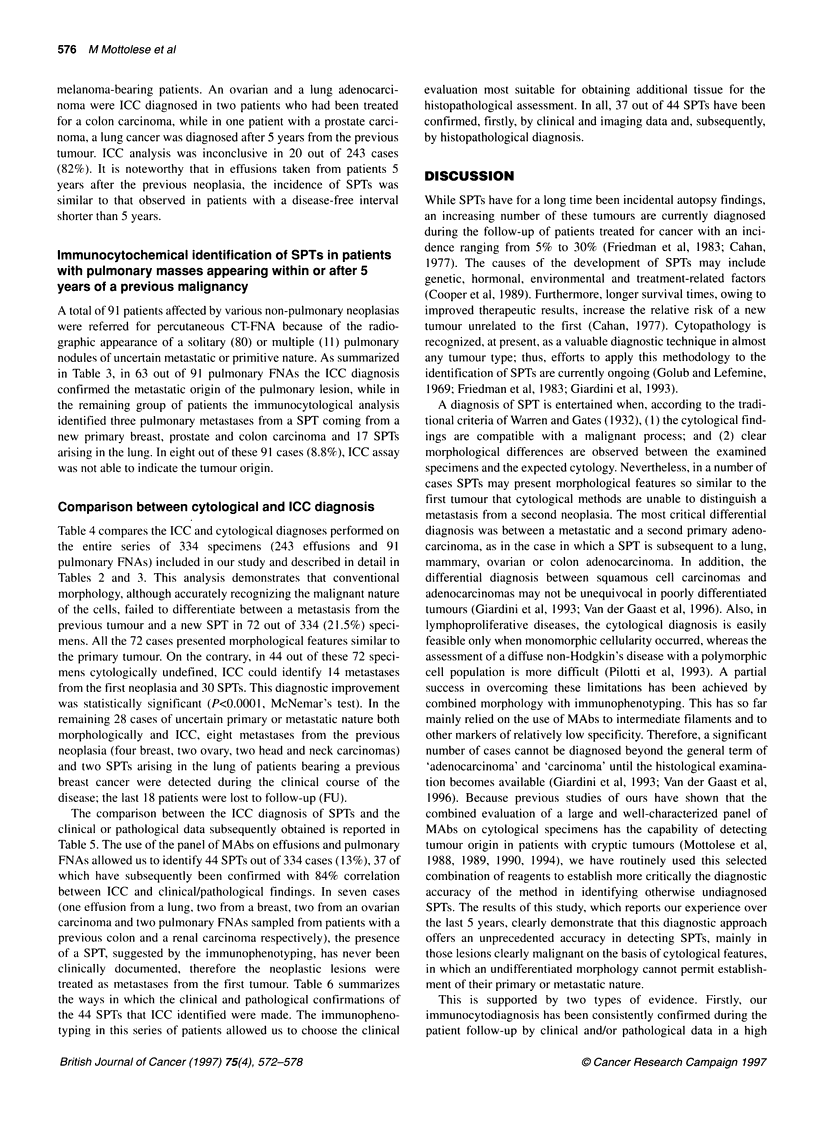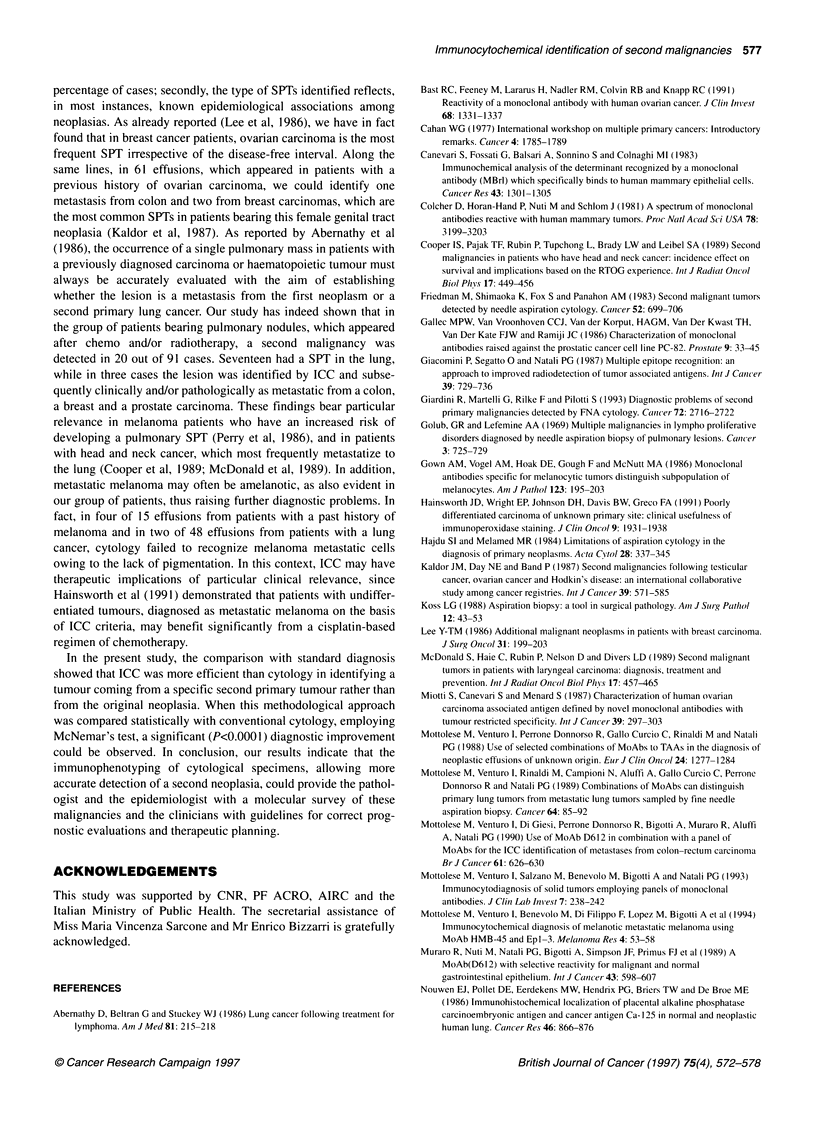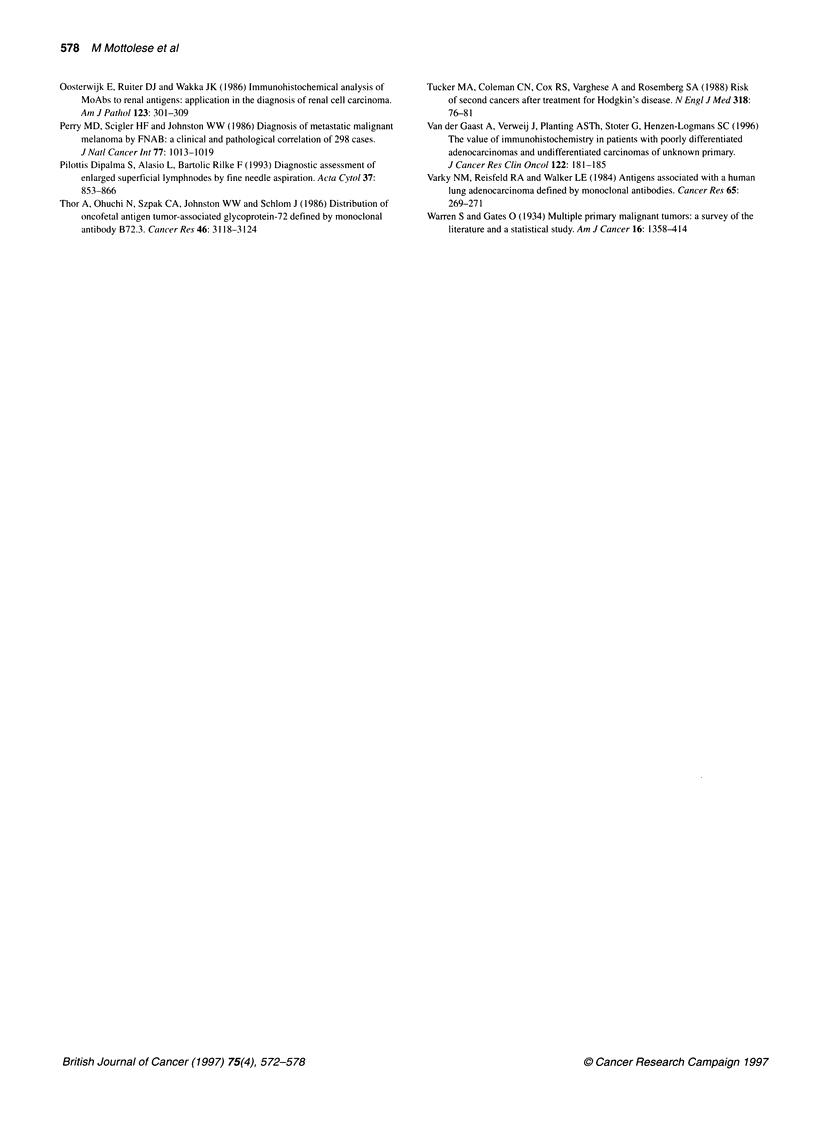# Identification of second malignancies on effusions and fine-needle aspirates using a panel of monoclonal antibodies.

**DOI:** 10.1038/bjc.1997.100

**Published:** 1997

**Authors:** M. Mottolese, I. Venturo, M. Rinaldi, M. Lopez, G. Bigotti, M. Benevolo, P. G. Natali

**Affiliations:** Regina Elena Cancer Institute, Rome, Italy.

## Abstract

The longer survival of neoplastic patients achieved through improvements of therapeutic regimens has increased the relative risk of developing a second primary tumour (SPT). In this context, conventional cytopathology can define tumour histotype only in a small fraction of cases. In this study, we have evaluated whether selected combinations of monoclonal antibodies (MAbs) to tumour-associated antigens (TAAs) can increase the accuracy of conventional morphology in detecting second primary tumours (SPTs) in two particularly difficult areas of cytodiagnosis, namely that of effusions and pulmonary fine-needle aspirates (FNAs). The immunocytochemical (ICC) analysis of 334 cytological specimens demonstrated that the use of our selected panel of MAbs could allow a more efficient identification of SPTs in comparison with conventional morphology. This diagnostic improvement was statistically significant (P < 0.0001). The present findings show that the immunophenotyping of effusions and FNAs, providing a more accurate and objective identification of SPTs, may have significant therapeutic and epidemiological relevance.


British Journal of Cancer (1997) 75(4), 572–578
© 1997 Cancer Research Campaign

### Identification of second malignancies on effusions and fine-needle aspirates using a panel of monoclonal antibodies

**M Mottolese[1], I Venturo[1], M Rinaldi[1], M Lopez[1], G Bigotti[2], M Benevolo[1] and PG Natali[1]**

[1]Regina Elena Cancer Institute, Viale Regina Elena 291, 00161 Rome; [2]Catholic University, Rome, Italy

**Summary** The longer survival of neoplastic patients achieved through improvements of therapeutic regimens has increased the relative risk of developing a second primary tumour (SPT). In this context, conventional cytopathology can define tumour histotype only in a small fraction of cases. In this study, we have evaluated whether selected combinations of monoclonal antibodies (MAbs) to tumour-associated antigens (TAAs) can increase the accuracy of conventional morphology in detecting second primary tumours (SPTs) in two particularly difficult areas of cytodiagnosis, namely that of effusions and pulmonary fine-needle aspirates (FNAs). The immunocytochemical (ICC) analysis of 334 cytological specimens demonstrated that the use of our selected panel of MAbs could allow a more efficient identification of SPTs in comparison with conventional morphology. This diagnostic improvement was statistically significant ($P<0.0001$). The present findings show that the immunophenotyping of effusions and FNAs, providing a more accurate and objective identification of SPTs, may have significant therapeutic and epidemiological relevance.

**Keywords:** second neoplasias; cytological diagnosis; immunocytochemistry; metastases

Second primary tumours (SPTs) have been diagnosed more frequently in recent years during the clinical course of patients bearing haematopoietic (Abernathy et al, 1986; Tucker et al, 1988) and solid tumours (Lee, 1986; Kaldor et al, 1987). As early diagnosis of SPTs has major therapeutic and epidemiological relevance (Kaldor et al, 1987; Giardini et al, 1993), the development of new methods for the accurate detection of second malignancies should be attempted. In this context, exfoliative and FNA cytology may be considered a valuable and accurate diagnostic technique, easy to perform on a large scale during the follow-up of patients previously treated for malignant tumours. These methods also have the advantage of minimal morbidity and low cost (Koss, 1988). While this approach has been reported to be highly accurate in identifying metastatic cells, the possibility of defining the tumour histotype has been far less successful (Friedman et al, 1983; Hajdu et al, 1984). We have previously reported that the use of selected combinations of MAbs to TAAs can be applied to a number of areas of cytodiagnosis of solid tumours, thus increasing the accuracy of conventional morphology in identifying metastases from unknown primary tumour and in differentiating primary from metastatic lesions (Mottolese et al, 1993). In the present study, we have analysed, in a large group of patients with a past history of malignancy, whether the use of a similar panel of reagents may also be useful in detecting SPTs in two areas of cytopathology, which are particularly difficult on the basis of morphological criteria, namely that of effusions and of pulmonary FNA.

*Received 4 April 1996*
*Revised 6 August 1996*
*Accepted 28 August 1996*

*Correspondence to:* PG Natali, Immunology Laboratory, Regina Elena Cancer Institute, Via delle Messi D'Oro, 156-00158 Rome, Italy

### MATERIALS AND METHODS

### Patients

From January 1990 to June 1995, 334 cytological specimens, of which 91 were pulmonary FNA and 243 pleural and peritoneal effusions sampled from patients previously treated with chemo and/or radiotherapy for different malignant tumours, were analysed both cytologically and immunocytochemically (ICC). The series consisted of 93 patients with effusions and 52 with solitary or multiple pulmonary masses, which appeared within 5 years of a previous tumour, while 189 patients developed effusions (150 cases) or radiologically assessed lung lesions (39 cases) at least 5 years after the first tumour. Only those cases in which morphological examination assessed the presence of malignant cells on cytological specimens have been included in this study. Unsatisfactory or insufficient specimens were excluded.

### Preparation of cell substrates

Pleural and peritoneal effusions were collected in sterile conditions using heparin (Liquemin Roche) as anticoagulant. Samples were centrifuged at 160 *g* for 10 min and the recovered cells were resuspended, after three washings with Hanks' balanced salt solution (Gibco Laboratories, Paisley, UK), at a density of $1 \times 10^6$ cells $ml^{-1}$. Red blood cells, when present, were removed by lysis with Tris/ammonium chloride pH 7.4, for 10 min at 37°C. Cytospins were obtained using a Shandon cytocentrifuge (Shandon, Runcorn, Cheshire, UK) and either stained according to the Papanicolau method for conventional morphological analysis or fixed in cold absolute acetone and immediately stored at –20°C for ICC evaluation. Pulmonary FNAs were performed under computerized tomography (CT) guidance with a 22-gauge needle placed on a 20-ml disposable syringe mounted

**Table 1** Antigenic phenotype of different solid tumours identified with a panel of MAbs to TAAs[a]

| MAbs | | | | | | | | | | Antigenic Phenotype | |
|---|---|---|---|---|---|---|---|---|---|---|---|
| B72.3 | B6.2 | MBrl | MOv 18–19 | OC-125 | KS1/4 | D612 | RC38 | PSA | Ep1–3 | | |
| + | + | + | – | – | NS[b] | – | – | – | – | Breast | carcinoma |
| + | – | – | + | + | NS[b] | – | – | – | – | Ovarian | carcinoma |
| + | – | – | – | + | + | – | – | – | – | Lung | carcinoma |
| + | – | – | – | – | – | + | – | – | – | Colon | carcinoma |
| + | – | – | – | – | – | – | + | – | – | Kidney | carcinoma |
| + | Ns[b] | – | – | – | NS[b] | – | – | + | – | Prostate | carcinoma |
| – | – | – | – | – | – | – | – | – | + | Melanoma | |

[a]See Mottolese et al (1988, 1989, 1990, 1993, 1994). [b] NS, not significant to detect tumour origin.

**Table 2** Immunocytochemical identification of SPTs in metastatic effusions from patients with a previous history of malignancy

| First tumour | No. of patients | Immunocytochemical diagnosis[a] | | |
|---|---|---|---|---|
| | | Metastasis from first tumour | Metastasis from SPTs | Undefined malignant tumour |
| Breast carcinoma | 95 | 74 | 8 Ovarian<br>3 Lung<br>2 Colon<br>1 Kidney<br>14 | 7 |
| Ovarian carcinoma | 61 | 52 | 2 Breast<br>1 Colon<br>3 | 6 |
| Lung carcinoma | 48 | 39 | 2 Melanoma | 7 |
| Colon carcinoma | 17 | 15 | 1 Ovarian<br>1 Lung<br>2 | |
| Melanoma | 15 | 13 | 2 Lung | |
| Prostate carcinoma | 7 | 6 | 1 Lung | |
| Total | 243 | 199 | 24 | 20 (8.2%) |

[a]Performed according to the pattern of reactivity summarized in Table 1.

on a special holder (Cameco 20 ml, Precision Dynamics, Burbank, CA, USA). Cellular specimens, smeared onto acid-clean glass slides, were fixed in 95% ethanol for conventional cytological diagnosis and in cold absolute acetone for ICC analysis.

### Monoclonal antibodies and immunocytochemical assays

In order to identify the primary tumour site, we selected a large panel of MAbs directed to different TAAs. For the purpose of this study, we classified the reagents on the basis of their main tumour specificity, as follows.

### *MAbs against breast cancer-associated antigens*

MAbs B72.3 and B6.2 were commercially obtained from Sorin Biomedica (Saluggia, Italy), while MAb MBrl was kindly provided by Professor MI Colnaghi (National Cancer Institute, Milan, Italy). MAb B72.3 identifies a high molecular weight glycoprotein expressed by the majority of adenocarcinomas and by 70% of breast carcinomas independent of histotype (Thor et al, 1986). MAb B6.2, which reacts with 80% of mammary adenocarcinomas, is also expressed by polymorpholeucocytes and by a high fraction of pulmonary and prostate carcinomas (Colcher et al, 1981). MAb MBrl recognizes a cell membrane neutral glycolipidic antigen, expressed by normal mammary epithelial cells, in about 70% of breast carcinomas and 40% of ovarian carcinomas (Canevari et al, 1983).

### *MAbs against ovarian cancer-associated antigens*

MAbs MOv18 and MOv19 were obtained from Professor MI Colnaghi (National Cancer Institute, Milan, Italy) and MAb OC-125 from Cis Diagnostici (Tronzano Vercellese, Italy). The first two reagents display a highly restricted tumour specificity with 85% of serous and endometrioid ovarian carcinomas (Miotti et al, 1987), while the glycoprotein Ca-125, expressed by 80% of non-mucinous ovarian epithelial malignant tumours (Bast et al, 1991), is also present in 70% of non-small-cell lung carcinomas and in normal bronchial epithelium (Nouwen et al, 1986). In our study this antigen was detectable on activated mesothelial cells in only a small percentage of pleural and peritoneal effusions.

© Cancer Research Campaign 1997

Table 3 Immunocytochemical identification of SPTs in pulmonary FNA of patients with a past history of malignancy

| First tumour | No. of patients | Immunocytochemical diagnosis[a] | | | |
|---|---|---|---|---|---|
| | | Metastasis from the first-tumour | Metastasis from a SPT | SPT[b] | Undefined |
| Breast carcinoma | 30 | 25 | | 3 | 2 |
| Ovarian carcinoma | 12 | 11 | 1[c] | | |
| Colon carcinoma | 6 | 4 | | 2 | |
| Melanoma | 9 | 6 | | 3 | |
| Head and neck carcinoma | 10 | – | 2[d] | 2 | 6 |
| Bladder carcinoma | 3 | 1 | | 2 | |
| Kidney carcinoma | 5 | 4 | | 1 | |
| NH lymphoma | 16 | 12 | | 4 | |
| Total | 91 | 63 | 3 | 17 | 8 (8.8%) |

[a]Performed according to the pattern of reactivity summarized in Table 1. [b]Lung carcinoma. [c]Breast carcinoma. [d]One prostate carcinoma and one colon carcinoma. NH, non-Hodgkin's.

Table 4 Comparison between diagnostic potential of cytology and immunocytochemistry in identifying SPTs in effusions and pulmonary FNAs

| First tumour | No. of patients | Metastasis | | Second tumour | | Undefined malignant tumour | |
|---|---|---|---|---|---|---|---|
| | | Cytology | ICC | Cytology | ICC | Cytology | ICC |
| Breast carcinoma | 125 | 97 | 99 | 7 | 17 | 21 | 9 |
| Ovarian carcinoma | 73 | 61 | 63 | 0 | 4 | 12 | 6 |
| Lung carcinoma | 48 | 37 | 39 | 0 | 2 | 11 | 7 |
| Colon carcinoma | 23 | 17 | 19 | 2 | 4 | 4 | 0 |
| Melanoma | 24 | 14 | 19 | 1 | 5 | 9 | 0 |
| Prostate carcinoma | 7 | 5 | 6 | 0 | 1 | 2 | 0 |
| Head and neck carcinoma | 10 | 1 | 0 | 0 | 4 | 9 | 6 |
| NH lymphoma | 16 | 11 | 12 | 3 | 4 | 2 | 0 |
| Kidney carcinoma | 5 | 4 | 4 | 0 | 1 | 1 | 0 |
| Bladder carcinoma | 3 | 1 | 1 | 1 | 2 | 1 | 0 |
| Total | 334 | 248 | 262[a] | 14 | 44[a] | 72 (21.5%) | 28 (8.3%) |

[a]The diagnostic improvement obtained through ICC analysis was statistically significant in comparison with standard cytology. $P<0.0001$ (McNemar's test). NH, non-Hodgkin's.

### *MAbs against lung, gastrointestinal, prostate and renal carcinomas*

MAb KS1/4, directed to a lung cancer-associated antigen, kindly provided by Dr Reisfeld (Scripps Clinic, La Jolla, CA, USA), reacts with 95% of lung carcinomas, including small-cell lung cancer, and stains epithelial alveolar cells faintly and heterogeneously (Varky et al, 1984).

MAb D612 identifies 85% of primary and metastatic gastrointestinal carcinomas and was obtained from Dr J Schlom (NHI, Bethesda, USA) (Muraro et al, 1989). MAbs LI838 (PSA) (Dakopatts, Copenhagen, Denmark) and RC38 (Unipath Spa, Italy) demonstrate a highly restricted tumour specificity for prostatic (95%) and renal (97%) carcinomas respectively (Gallec et al, 1986, Oosterwijk et al, 1986).

### *MAbs against melanoma-associated antigens.*

MAbs HMB45 (Gown et al, 1986) and Ep1–3 (Giacomini et al, 1987) were obtained from Dako and Immunology Laboratory, Regina Elena Cancer Institute, Rome, Italy respectively. MAb Ep1–3, which recognizes a high molecular weight melanoma-associated antigen (HMW-MAA), is made up by mixing equimolar concentrations of three reagents, Ep1, Ep2, Ep3. This pool of reagents demonstrates a restricted reactivity with both primary and metastatic melanoma, and also in amelanotic cells.

### *MAbs to intermediate filaments and lymphoid antigens*

Additional ICC stainings were performed, in some instances, employing MAbs anti-paqcytokeratins (MNF116), anti-human leucocyte common antigen (CD45), anti-T cell (CD3 and CD45RO) and anti-B cell (CD45RA and CD20), purchased from Dako.

The pattern of reactivity of these reagents is described in Table 1. The use of multiple MAbs, as we previously demonstrated on a large number of different cytological specimens, is imposed by the lack of absolute tumour specificity of the selected reagents and by the need to overcome the heterogeneous expression of TAAs, which may often result in false-negative findings. (Mottolese et al, 1988, 1989, 1993, 1994).

The immunoreactivity of the MAbs was controlled repeatedly during the study using negative and positive control specimens. Cytological preparations were stained employing a sensitive biotin–streptavidin–immunoperoxidase method (LSAB Kit, Dako), and the enzymatic activity was developed with 3-amino-9-ethylcarbazole using Mayer's haematoxylin as nuclear counterstaining. Slides were mounted in aqueous mounting medium (Glycergel, Dako). The ICC findings were evaluated independently by two investigators who had no knowledge of the cytopathological diagnosis. The cytological and immunocytochemical findings were compared with those obtained by histopathology and/or with the clinical data obtained during patient follow-up.

 © Cancer Research Campaign 1997

**Table 5** Comparison between ICC diagnoses of SPTs and clinical and pathological data obtained during the patient follow-up

| First tumour | No. of patients | No. of SPTs ICC diagnosed | Confirmed diagnoses by clinical and pathological data |
|---|---|---|---|
| Breast carcinoma | 125 | 17 | 15 |
| Ovarian carcinoma | 73 | 4 | 2 |
| Lung carcinoma | 48 | 2 | 1 |
| Colon carcinoma | 23 | 4 | 3 |
| Melanoma | 24 | 5 | 5 |
| Prostate carcinoma | 7 | 1 | 1 |
| Head and neck carcinoma | 10 | 4 | 4 |
| Bladder carcinoma | 3 | 2 | 2 |
| NH lymphoma | 16 | 4 | 4 |
| Kidney carcinoma | 5 | 1 | 0 |
| Total | 334 | 44 | 37[a] |

[a]84% correlation with ICC diagnosis.

### RESULTS

### Immunocytochemical identification of SPTs in metastatic effusions from patients with a past history of malignancy

A total of 243 pleural or peritoneal effusions, which appeared in patients with a past history of malignancy, were analysed ICC using the panel of MAbs described in Table 1. This combination of reagents, in which both positive and negative immunoreactivities bear diagnostic information, identifies distinct antigenic phenotypes that can help to discriminate solid tumours of most common occurrence. As summarized in Table 2, 199 of the 243 effusions were defined, on the basis of their immunophenotype, as metastatic from the first tumour. In 24 patients, on the other hand, immunocytology suggested the presence of a metastasis from a SPT. Fourteen of these 24 patients had previously been treated for breast cancer, and the second malignancy originated in the ovary (eight cases), in the lung (three cases), in the colon (two cases) and in the kidney (one case). Three patients had a past history of ovarian carcinoma, two of whom developed a mammary and a colon carcinoma respectively. A melanoma appeared in two patients with a lung carcinoma, and a lung cancer in two

**Table 6** Clinical and pathological confirmations of the SPTs immunocytochemically diagnosed

| First tumour | Clinical presentation of disease | No. of cases | ICC diagnoses | Subsequent clinical evaluation | No. of confirmed ICC diagnoses | Histological diagnoses |
|---|---|---|---|---|---|---|
| Breast carcinoma | Pleural effusion | 3 | Lung cancer | Bronchoscopy + biopsy | 2 | 1 Adc + 1 undifferentiated carcinoma |
| Breast carcinoma | Peritoneal effusion | 8 | Ovarian cancer | Laparoscopy + biopsy | 7 | 5 Serous adc +2 undifferentiated carcinoma |
| Breast carcinoma | Peritoneal effusion | 2 | Colon cancer | Colonoscopy + biopsy | 2 | 2 Undifferentiated adc |
| Breast carcinoma | Peritoneal effusion | 1 | Renal cancer | CT scan + biopsy | 1 | 1 Renal cell carcinoma |
| Breast carcinoma | Pulmonary mass | 3 | Lung cancer | Bronchoscopy + biopsy | 3 | 2 Adc + 1 undifferentiated carcinoma |
| | | 17 | | | 15 | |
| Ovarian carcinoma | Pleural effusion | 2 | Breast cancer | Mammography + FNA | 1 | 1 Ductal carcinoma |
| Ovarian carcinoma | Peritoneal effusion | 1 | Colon cancer | Colonoscopy + biopsy | 1 | 1 Undifferentiated adc |
| Ovarian carcinoma | Pulmonary mass | 1 | Lung cancer | Bronchoscopy + biopsy | Not confirmed | Metastatic ovarian carcinoma |
| | | 4 | | | 2 | |
| Lung carcinoma | Pleural effusion | 2 | Melanoma | Dermatological exam + surgery | 1 | 1 Nodular melanoma (IV Clark lev) |
| Colon carcinoma | Pleural effusion | 1 | Lung cancer | Bronchoscopy + biopsy | 1 | 1 Undifferentiated adc |
| Colon carcinoma | Peritoneal effusion | 1 | Ovarian cancer | Laparoscopy + biopsy | 1 | 1 Serous adc |
| Colon carcinoma | Pulmonary mass | 2 | Lung cancer | Bronchoscopy + biopsy | 1 | 1 Undifferentiated carcinoma |
| | | 4 | | | 3 | |
| Melanoma | Pleural effusion | 2 | Lung cancer | Bronchoscopy + biopsy | 2 | 2 Undifferentiated carcinoma |
| Melanoma | Pulmonary mass | 3 | Lung cancer | Bronchoscopy + biopsy | 3 | 1 Adc + 2 undifferentiated carcinoma |
| | | 5 | | | 5 | |
| Head and neck carcinoma | Pulmonary mass | 2 | Lung cancer | Bronchoscopy + biopsy | 2 | 2 Undifferentiated carcinoma |
| Head and neck carcinoma | Pulmonary mass | 1 | Colon cancer | Colonoscopy + biopsy | 1 | 1 Undifferentiated carcinoma |
| Head and neck carcinoma | Pulmonary mass | 1 | Prostate cancer | Transrectal ecotom. + biopsy | 1 | 1 Adc |
| | | 4 | | | 4 | |
| Prostate carcinoma | Pleural effusion | 1 | Lung cancer | Bronchoscopy + biopsy | 1 | 1 Undifferentiated carcinoma |
| Bladder carcinoma | Pulmonary mass | 2 | Lung cancer | Bronchoscopy + biopsy | 2 | 1 Adc + 1 undifferentiated carcinoma |
| Renal carcinoma | Pulmonary mass | 1 | Lung cancer | Bronchoscopy + biopsy | Not confirmed | Metastatic renal cell carcinoma |
| NHLymphoma | Pulmonary mass | 4 | Lung cancer | Bronchoscopy + biopsy | 4 | 2 Undifferentiated adc + 2 undifferentiated carcinoma |
| Total | | 44 | | | 37 | |

NHL, non-Hodgkin's lymphoma. Adc, adenocarcinoma.

*© Cancer Research Campaign 1997*

melanoma-bearing patients. An ovarian and a lung adenocarcinoma were ICC diagnosed in two patients who had been treated for a colon carcinoma, while in one patient with a prostate carcinoma, a lung cancer was diagnosed after 5 years from the previous tumour. ICC analysis was inconclusive in 20 out of 243 cases (82%). It is noteworthy that in effusions taken from patients 5 years after the previous neoplasia, the incidence of SPTs was similar to that observed in patients with a disease-free interval shorter than 5 years.

### Immunocytochemical identification of SPTs in patients with pulmonary masses appearing within or after 5 years of a previous malignancy

A total of 91 patients affected by various non-pulmonary neoplasias were referred for percutaneous CT-FNA because of the radiographic appearance of a solitary (80) or multiple (11) pulmonary nodules of uncertain metastatic or primitive nature. As summarized in Table 3, in 63 out of 91 pulmonary FNAs the ICC diagnosis confirmed the metastatic origin of the pulmonary lesion, while in the remaining group of patients the immunocytological analysis identified three pulmonary metastases from a SPT coming from a new primary breast, prostate and colon carcinoma and 17 SPTs arising in the lung. In eight out of these 91 cases (8.8%), ICC assay was not able to indicate the tumour origin.

### Comparison between cytological and ICC diagnosis

Table 4 compares the ICC and cytological diagnoses performed on the entire series of 334 specimens (243 effusions and 91 pulmonary FNAs) included in our study and described in detail in Tables 2 and 3. This analysis demonstrates that conventional morphology, although accurately recognizing the malignant nature of the cells, failed to differentiate between a metastasis from the previous tumour and a new SPT in 72 out of 334 (21.5%) specimens. All the 72 cases presented morphological features similar to the primary tumour. On the contrary, in 44 out of these 72 specimens cytologically undefined, ICC could identify 14 metastases from the first neoplasia and 30 SPTs. This diagnostic improvement was statistically significant ($P<0.0001$, McNemar's test). In the remaining 28 cases of uncertain primary or metastatic nature both morphologically and ICC, eight metastases from the previous neoplasia (four breast, two ovary, two head and neck carcinomas) and two SPTs arising in the lung of patients bearing a previous breast cancer were detected during the clinical course of the disease; the last 18 patients were lost to follow-up (FU).

The comparison between the ICC diagnosis of SPTs and the clinical or pathological data subsequently obtained is reported in Table 5. The use of the panel of MAbs on effusions and pulmonary FNAs allowed us to identify 44 SPTs out of 334 cases (13%), 37 of which have subsequently been confirmed with 84% correlation between ICC and clinical/pathological findings. In seven cases (one effusion from a lung, two from a breast, two from an ovarian carcinoma and two pulmonary FNAs sampled from patients with a previous colon and a renal carcinoma respectively), the presence of a SPT, suggested by the immunophenotyping, has never been clinically documented, therefore the neoplastic lesions were treated as metastases from the first tumour. Table 6 summarizes the ways in which the clinical and pathological confirmations of the 44 SPTs that ICC identified were made. The immunophenotyping in this series of patients allowed us to choose the clinical evaluation most suitable for obtaining additional tissue for the histopathological assessment. In all, 37 out of 44 SPTs have been confirmed, firstly, by clinical and imaging data and, subsequently, by histopathological diagnosis.

### DISCUSSION

While SPTs have for a long time been incidental autopsy findings, an increasing number of these tumours are currently diagnosed during the follow-up of patients treated for cancer with an incidence ranging from 5% to 30% (Friedman et al, 1983; Cahan, 1977). The causes of the development of SPTs may include genetic, hormonal, environmental and treatment-related factors (Cooper et al, 1989). Furthermore, longer survival times, owing to improved therapeutic results, increase the relative risk of a new tumour unrelated to the first (Cahan, 1977). Cytopathology is recognized, at present, as a valuable diagnostic technique in almost any tumour type; thus, efforts to apply this methodology to the identification of SPTs are currently ongoing (Golub and Lefemine, 1969; Friedman et al, 1983; Giardini et al, 1993).

A diagnosis of SPT is entertained when, according to the traditional criteria of Warren and Gates (1932), (1) the cytological findings are compatible with a malignant process; and (2) clear morphological differences are observed between the examined specimens and the expected cytology. Nevertheless, in a number of cases SPTs may present morphological features so similar to the first tumour that cytological methods are unable to distinguish a metastasis from a second neoplasia. The most critical differential diagnosis was between a metastatic and a second primary adenocarcinoma, as in the case in which a SPT is subsequent to a lung, mammary, ovarian or colon adenocarcinoma. In addition, the differential diagnosis between squamous cell carcinomas and adenocarcinomas may not be unequivocal in poorly differentiated tumours (Giardini et al, 1993; Van der Gaast et al, 1996). Also, in lymphoproliferative diseases, the cytological diagnosis is easily feasible only when monomorphic cellularity occurred, whereas the assessment of a diffuse non-Hodgkin's disease with a polymorphic cell population is more difficult (Pilotti et al, 1993). A partial success in overcoming these limitations has been achieved by combined morphology with immunophenotyping. This has so far mainly relied on the use of MAbs to intermediate filaments and to other markers of relatively low specificity. Therefore, a significant number of cases cannot be diagnosed beyond the general term of 'adenocarcinoma' and 'carcinoma' until the histological examination becomes available (Giardini et al, 1993; Van der Gaast et al, 1996). Because previous studies of ours have shown that the combined evaluation of a large and well-characterized panel of MAbs on cytological specimens has the capability of detecting tumour origin in patients with cryptic tumours (Mottolese et al, 1988, 1989, 1990, 1994), we have routinely used this selected combination of reagents to establish more critically the diagnostic accuracy of the method in identifying otherwise undiagnosed SPTs. The results of this study, which reports our experience over the last 5 years, clearly demonstrate that this diagnostic approach offers an unprecedented accuracy in detecting SPTs, mainly in those lesions clearly malignant on the basis of cytological features, in which an undifferentiated morphology cannot permit establishment of their primary or metastatic nature.

This is supported by two types of evidence. Firstly, our immunocytodiagnosis has been consistently confirmed during the patient follow-up by clinical and/or pathological data in a high

© Cancer Research Campaign 1997

percentage of cases; secondly, the type of SPTs identified reflects, in most instances, known epidemiological associations among neoplasias. As already reported (Lee et al, 1986), we have in fact found that in breast cancer patients, ovarian carcinoma is the most frequent SPT irrespective of the disease-free interval. Along the same lines, in 61 effusions, which appeared in patients with a previous history of ovarian carcinoma, we could identify one metastasis from colon and two from breast carcinomas, which are the most common SPTs in patients bearing this female genital tract neoplasia (Kaldor et al, 1987). As reported by Abernathy et al (1986), the occurrence of a single pulmonary mass in patients with a previously diagnosed carcinoma or haematopoietic tumour must always be accurately evaluated with the aim of establishing whether the lesion is a metastasis from the first neoplasm or a second primary lung cancer. Our study has indeed shown that in the group of patients bearing pulmonary nodules, which appeared after chemo and/or radiotherapy, a second malignancy was detected in 20 out of 91 cases. Seventeen had a SPT in the lung, while in three cases the lesion was identified by ICC and subsequently clinically and/or pathologically as metastatic from a colon, a breast and a prostate carcinoma. These findings bear particular relevance in melanoma patients who have an increased risk of developing a pulmonary SPT (Perry et al, 1986), and in patients with head and neck cancer, which most frequently metastatize to the lung (Cooper et al, 1989; McDonald et al, 1989). In addition, metastatic melanoma may often be amelanotic, as also evident in our group of patients, thus raising further diagnostic problems. In fact, in four of 15 effusions from patients with a past history of melanoma and in two of 48 effusions from patients with a lung cancer, cytology failed to recognize melanoma metastatic cells owing to the lack of pigmentation. In this context, ICC may have therapeutic implications of particular clinical relevance, since Hainsworth et al (1991) demonstrated that patients with undifferentiated tumours, diagnosed as metastatic melanoma on the basis of ICC criteria, may benefit significantly from a cisplatin-based regimen of chemotherapy.

In the present study, the comparison with standard diagnosis showed that ICC was more efficient than cytology in identifying a tumour coming from a specific second primary tumour rather than from the original neoplasia. When this methodological approach was compared statistically with conventional cytology, employing McNemar's test, a significant ($P<0.0001$) diagnostic improvement could be observed. In conclusion, our results indicate that the immunophenotyping of cytological specimens, allowing more accurate detection of a second neoplasia, could provide the pathologist and the epidemiologist with a molecular survey of these malignancies and the clinicians with guidelines for correct prognostic evaluations and therapeutic planning.

### ACKNOWLEDGEMENTS

This study was supported by CNR, PF ACRO, AIRC and the Italian Ministry of Public Health. The secretarial assistance of Miss Maria Vincenza Sarcone and Mr Enrico Bizzarri is gratefully acknowledged.

### REFERENCES

Abernathy D, Beltran G and Stuckey WJ (1986) Lung cancer following treatment for lymphoma. *Am J Med* **81**: 215–218

Bast RC, Feeney M, Lararus H, Nadler RM, Colvin RB and Knapp RC (1991) Reactivity of a monoclonal antibody with human ovarian cancer. *J Clin Invest* **68**: 1331–1337

Cahan WG (1977) International workshop on multiple primary cancers: Introductory remarks. *Cancer* **4**: 1785–1789

Canevari S, Fossati G, Balsari A, Sonnino S and Colnaghi MI (1983) Immunochemical analysis of the determinant recognized by a monoclonal antibody (MBrl) which specifically binds to human mammary epithelial cells. *Cancer Res* **43**: 1301–1305

Colcher D, Horan-Hand P, Nuti M and Schlom J (1981) A spectrum of monoclonal antibodies reactive with human mammary tumors. *Proc Natl Acad Sci USA* **78**: 3199–3203

Cooper IS, Pajak TF, Rubin P, Tupchong L, Brady LW and Leibel SA (1989) Second malignancies in patients who have head and neck cancer: incidence effect on survival and implications based on the RTOG experience. *Int J Radiat Oncol Biol Phys* **17**: 449–456

Friedman M, Shimaoka K, Fox S and Panahon AM (1983) Second malignant tumors detected by needle aspiration cytology. *Cancer* **52**: 699–706

Gallec MPW, Van Vroonhoven CCJ, Van der Korput, HAGM, Van Der Kwast TH, Van Der Kate FJW and Ramiji JC (1986) Characterization of monoclonal antibodies raised against the prostatic cancer cell line PC-82. *Prostate* **9**: 33–45

Giacomini P, Segatto O and Natali PG (1987) Multiple epitope recognition: an approach to improved radiodetection of tumor associated antigens. *Int J Cancer* **39**: 729–736

Giardini R, Martelli G, Rilke F and Pilotti S (1993) Diagnostic problems of second primary malignancies detected by FNA cytology. *Cancer* **72**: 2716–2722

Golub, GR and Lefemine AA (1969) Multiple malignancies in lympho proliferative disorders diagnosed by needle aspiration biopsy of pulmonary lesions. *Cancer* **3**: 725–729

Gown AM, Vogel AM, Hoak DE, Gough F and McNutt MA (1986) Monoclonal antibodies specific for melanocytic tumors distinguish subpopulation of melanocytes. *Am J Pathol* **123**: 195–203

Hainsworth JD, Wright EP, Johnson DH, Davis BW, Greco FA (1991) Poorly differentiated carcinoma of unknown primary site: clinical usefulness of immunoperoxidase staining. *J Clin Oncol* **9**: 1931–1938

Hajdu SI and Melamed MR (1984) Limitations of aspiration cytology in the diagnosis of primary neoplasms. *Acta Cytol* **28**: 337–345

Kaldor JM, Day NE and Band P (1987) Second malignancies following testicular cancer, ovarian cancer and Hodkin's disease: an international collaborative study among cancer registries. *Int J Cancer* **39**: 571–585

Koss LG (1988) Aspiration biopsy: a tool in surgical pathology. *Am J Surg Pathol* **12**: 43–53

Lee Y-TM (1986) Additional malignant neoplasms in patients with breast carcinoma. *J Surg Oncol* **31**: 199–203

McDonald S, Haie C, Rubin P, Nelson D and Divers LD (1989) Second malignant tumors in patients with laryngeal carcinoma: diagnosis, treatment and prevention. *Int J Radiat Oncol Biol Phys* **17**: 457–465

Miotti S, Canevari S and Menard S (1987) Characterization of human ovarian carcinoma associated antigen defined by novel monoclonal antibodies with tumour restricted specificity. *Int J Cancer* **39**: 297–303

Mottolese M, Venturo I, Perrone Donnorso R, Gallo Curcio C, Rinaldi M and Natali PG (1988) Use of selected combinations of MoAbs to TAAs in the diagnosis of neoplastic effusions of unknown origin. *Eur J Clin Oncol* **24**: 1277–1284

Mottolese M, Venturo I, Rinaldi M, Campioni N, Aluffi A, Gallo Curcio C, Perrone Donnorso R and Natali PG (1989) Combinations of MoAbs can distinguish primary lung tumors from metastatic lung tumors sampled by fine needle aspiration biopsy. *Cancer* **64**: 85–92

Mottolese M, Venturo I, Di Giesi, Perrone Donnorso R, Bigotti A, Muraro R, Aluffi A, Natali PG (1990) Use of MoAb D612 in combination with a panel of MoAbs for the ICC identification of metastases from colon–rectum carcinoma *Br J Cancer* **61**: 626–630

Mottolese M, Venturo I, Salzano M, Benevolo M, Bigotti A and Natali PG (1993) Immunocytodiagnosis of solid tumors employing panels of monoclonal antibodies. *J Clin Lab Invest* **7**: 238–242

Mottolese M, Venturo I, Benevolo M, Di Filippo F, Lopez M, Bigotti A et al (1994) Immunocytochemical diagnosis of melanotic metastatic melanoma using MoAb HMB-45 and Ep1-3. *Melanoma Res* **4**: 53–58

Muraro R, Nuti M, Natali PG, Bigotti A, Simpson JF, Primus FJ et al (1989) A MoAb(D612) with selective reactivity for malignant and normal gastrointestinal epithelium. *Int J Cancer* **43**: 598–607

Nouwen EJ, Pollet DE, Eerdekens MW, Hendrix PG, Briers TW and De Broe ME (1986) Immunohistochemical localization of placental alkaline phosphatase carcinoembryonic antigen and cancer antigen Ca-125 in normal and neoplastic human lung. *Cancer Res* **46**: 866–876

© Cancer Research Campaign 1997

Oosterwijk E, Ruiter DJ and Wakka JK (1986) Immunohistochemical analysis of MoAbs to renal antigens: application in the diagnosis of renal cell carcinoma. *Am J Pathol* **123**: 301–309

Perry MD, Scigler HF and Johnston WW (1986) Diagnosis of metastatic malignant melanoma by FNAB: a clinical and pathological correlation of 298 cases. *J Natl Cancer Int* **77**: 1013–1019

Pilottis Dipalma S, Alasio L, Bartolic Rilke F (1993) Diagnostic assessment of enlarged superficial lymphnodes by fine needle aspiration. *Acta Cytol* **37**: 853–866

Thor A, Ohuchi N, Szpak CA, Johnston WW and Schlom J (1986) Distribution of oncofetal antigen tumor-associated glycoprotein-72 defined by monoclonal antibody B72.3. *Cancer Res* **46**: 3118–3124

Tucker MA, Coleman CN, Cox RS, Varghese A and Rosemberg SA (1988) Risk of second cancers after treatment for Hodgkin's disease. *N Engl J Med* **318**: 76–81

Van der Gaast A, Verweij J, Planting ASTh, Stoter G, Henzen-Logmans SC (1996) The value of immunohistochemistry in patients with poorly differentiated adenocarcinomas and undifferentiated carcinomas of unknown primary. *J Cancer Res Clin Oncol* **122**: 181–185

Varky NM, Reisfeld RA and Walker LE (1984) Antigens associated with a human lung adenocarcinoma defined by monoclonal antibodies. *Cancer Res* **65**: 269–271

Warren S and Gates O (1934) Multiple primary malignant tumors: a survey of the literature and a statistical study. *Am J Cancer* **16**: 1358–414

© Cancer Research Campaign 1997